# Association between inpatient glycemic variability and COVID-19 mortality: a prospective study

**DOI:** 10.1186/s13098-023-01157-z

**Published:** 2023-09-11

**Authors:** Salma Ali El Chab Parolin, Rebecca Benicio Stocco, Julia do Carmo Kneipp Lopes, Marcos Roberto Curcio Pereira, Milena Massae Yamashita, Maria Eduarda Domareski Goulart, Henrique Demeneck, Marcia Olandoski, Larissa Hermann de Souza Nunes, Victor Keniche Morisawa, Luiz Augusto Fanhani Cracco, Isabela Busto Silva, Jarbas Silva Motta Júnior, Daniela Veit Barreto, Gustavo Lenci Marques, Thyago Proença de Moraes, Cristina Pellegrino Baena

**Affiliations:** Pontifical Catholic University of Paraná (PUCPR), Rua Imaculada Conceição, 1155, Curitiba, Paraná 80215-901 Brazil

**Keywords:** COVID-19, Glycemic variability, Mortality, inpatient

## Abstract

**Background:**

This study aimed to determine the association between glycemic variability (GV) and mortality in hospitalized patients with coronavirus disease 2019 (COVID-19).

**Methods:**

We prospectively analyzed data from inpatients (> 18 years old) with RT-PCR confirmed COVID-19 admitted between March 2020 and July 2021. All patients were hospitalized for more than 48 h and had at least six point-of-care capillary glucose tests obtained three times daily in the pre-prandial period during hospitalization. GV was measured using the glucose standard deviation (SD) and coefficient of variation (CV). ROC curve was adjusted to determine the SD and CV cutoff values associated with mortality (44.7 mg/dL and 27.5%, respectively); values above these were considered indicative of high GV. Logistic regression models were fitted to explore the association between GV and mortality in patients with and without diabetes.

**Results:**

A total of 628 patients were stratified into SD < 44.7 mg/dL (n = 357) versus ≥ 44.7 mg/dL (n = 271) and CV < 27.5% (n = 318) versus ≥ 27.5% (n = 310) groups. After controlling for age, sex, presence of diabetes mellitus (DM) and cardiovascular disease, we found a significant association between high GV and mortality (odds ratio 2.99 [1.88–4.77] for SD and 2.43 [1.54–3.85] for CV; p values < 0.001). The mortality rate was higher with SD ≥ 44.7 mg/dL and CV ≥ 27.5% compared to that with SD < 44.7 mg/dL and CV < 27.5%, regardless of DM (p < 0.001 for all).

**Conclusion:**

High glycemic variability was independently associated with mortality in patients with and without DM, who were hospitalized with COVID-19.

## Introduction

Glucose variability (GV) is common among hospitalized patients, and the benefits of glycemic control have been demonstrated in acute infections regardless of the diagnosis of diabetes mellitus (DM). However, the role of GV in hospitalized patients with coronavirus disease 2019 (COVID-19) is not clear, and its prognostic implications are not understood.

Since December 2019, COVID-19 caused by a novel coronavirus, the severe acute respiratory syndrome coronavirus 2 (SARS-CoV-2) [1, 2], has been associated with substantial mortality. By January 2023, the number of deaths due to COVID-19 had exceeded 6.7 million worldwide. Hyperglycemia and DM are responsible for an increased risk of hospital admission, acute kidney injury, need for mechanical ventilation, intensive care unit (ICU) admission, and mortality in patients with COVID-19 [3]; however, the mechanisms underlying the increased risk associated with hyperglycemia and DM remain unclear [4].

Findings from retrospective studies have shown that high glycemic variability is associated with a longer length of hospital stay and increased mortality in patients hospitalized with or without acute infections, independent of the presence of DM [5, 6]. High glycemic variability is thought to boost oxidative stress, causing a poor T cell response and leading to severe COVID-19 manifestations. Indeed, Monnier et al. demonstrated in a case–control study that glucose fluctuations trigger oxidative stress, which is not observed in chronic sustained hyperglycemia [7].

Glycemic variability is a complex process that reflects blood glucose fluctuations occurring over a day or over a period of time, including periods of hypoglycemia and hyperglycemia. High glycemic variability is considered indicative of poor glycemic control [8]. Several metrics can be used to evaluate glycemic variability, and glucose standard deviation (SD) and coefficient of variation (CV) are considered simple and accurate methods for this purpose [9].

Based on these considerations, this study aimed to test the association between glycemic variability and mortality in patients with and without DM, hospitalized with confirmed COVID-19, to provide insights for care improvement in this population. We hypothesized a higher VG could be a risk factor for mortality and other poor outcomes during hospitalization in patients with COVID-19.

## Methods

### Study design and participants

This study using real-world data was conducted between March 2020 and July 2021 at a tertiary referral center (Hospital Marcelino Champagnat) in Brazil.

**Fig. 1:**
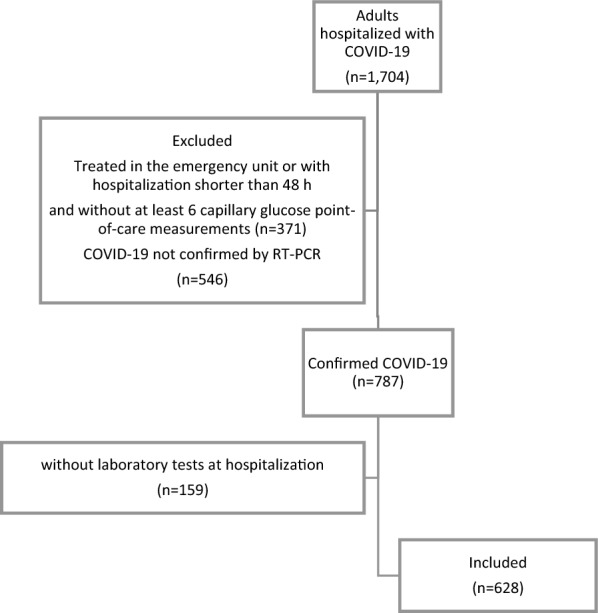
Flowchart of the participants' selection process. COVID-19: coronavirus disease-2019

All patients were included sequentially, were older than 18 years, had COVID-19 confirmed using reverse transcription polymerase chain reaction (RT-PCR) testing, were hospitalized for more than 48 h, and had at least six point-of-care capillary glucose tests (POCTs) obtained three times daily in the pre-prandial period during hospitalization (Fig. 1).

We used each patient's POCT results during the hospital stay to calculate individual SD and CV values. For patients hospitalized for > 10 days, we considered only the POCT results from the first 10 days to evaluate the interference of early glycemic variability in the outcome mortality.

We analyzed the cohort according to (1) SD values, as < 44.7 mg/dL versus ≥ 44.7 mg/dL, (2) CV values, as < 27.5% versus ≥ 27.5%, and (3) previous diagnosis of DM, as present versus absent. In the latter, we reanalyzed all parameters according to the presence or absence of DM to identify whether DM could have influenced mortality.

### Data collection

Data regarding patients' demographic characteristics (sex and age), comorbidities (hypertension; DM; cerebrovascular disease; chronic renal failure; obesity, defined as a body mass index ≥ 30 kg/m^2^; malignancy; dyslipidemia; cardiovascular disease [CVD]; and chronic respiratory diseases), use of corticosteroids before hospitalization, and medications used at home were collected in real time using a Philips Tasy electronic medical record (Philips Healthcare, Cambridge, MA, USA). A prior diagnosis of DM was considered to be present when reported by the patient or when the use of an oral hypoglycemic agent was reported on admission.

Data collection was monitored in real time by the authors. Laboratory data (results from COVID-19 RT-PCR tests, white blood cell count [cells/μL], lymphocytes [cells/μL], platelets [cells/μL], C-reactive protein [CRP; mg/L], creatinine [mg/dL], and neutrophil-to-lymphocyte ratio [N/L]) were obtained at admission and during hospitalization.

Glucose values were based on bedside POCT measurements of capillary blood obtained three times daily in the pre-prandial period, according to the hospital's protocol. The glucometer used was an Abbott FreeStyle Optium Neo (Abbott Diabetes Care Ltd., Witney, UK). Hypoglycemia was defined as capillary glucose concentration < 70 mg/dL (< 3.9 mmol/L).

The study protocol was approved by the hospital's research ethics committee (Center for Teaching, Research, and Innovation [CEPI]) on March 20, 2020, and was registered at Plataforma Brasil (CAAE 30188020.7.1001.0020). All patients or their legally authorized representatives signed consent forms before the start of the study. The study was conducted in accordance with the principles of the Declaration of Helsinki.

### Outcome

The primary study endpoint was the association of glycemic variability and mortality (assessed between hospital admission and discharge) among the patients hospitalized with COVID-19. Death data were obtained from the medical records.

### Sliding scale insulin therapy

The institutional protocol was followed for the adjustment of glucose levels to above 140 mg/dL using rapid-acting insulin. For critically ill patients using corticosteroids, the protocol recommends starting with 6 units of rapid-acting insulin and adding 2 units for each 40 mg/dL above the target glucose level. For noncritical patients, the protocol started with 4 units of rapid-acting insulin and 2 units increase for each 40 mg/dL above the target glucose level.

### Statistical analyses

Categorical variables were presented as numbers (%, maximum and minimum values). Continuous variables with a normal distribution were presented as mean (standard deviation [SD]), while those without a normal distribution were presented as medians (interquartile range [IQR]). The chi-square test was used to compare high versus low glycemic variability in patients with and without DM. Continuous variables were compared using the Mann–Whitney or Student's *t* test.

The association between mortality and age, sex, comorbidities, and use versus no use of corticosteroids during hospitalization was estimated using univariate and multivariate regression analyses, and the results were presented as odds ratios (ORs) and 95% confidence intervals (CIs). A receiver operating characteristic (ROC) curve was used to identify the cutoff values for SD and CV associated with mortality. Statistical significance was set at p < 0.05. All statistical analyses were performed using SPSS, version 28.0 (IBM Corp., Armonk, NY, USA).

## Results

In total, 1704 patients were hospitalized with COVID-19 between March 2020 and July 2021. We excluded patients who remained in the emergency department or were admitted to the hospital for less than 48 h, those with fewer than six POCT measurements during hospitalization (N = 371), and those without RT-PCR confirmation of COVID-19 (n = 546) and admission laboratory tests (n = 159). The final sample included 628 patients admitted to the hospital for more than 48 h, with at least six glucose POCTs and confirmed COVID-19 (Fig. 1).

The mean age of the included patients was 59.6 ± 15.9 years, and 65.8% were men. The most prevalent comorbidities were hypertension and DM (Table 1).

**Table 1:** General characteristics of the study population (n = 628)

Characteristics	Values
Age (mean ± SD)	59.62 ± 15.899
Male gender	413 (65.8%)
Comorbidities	
Hypertension	328 (52.2%)
Diabetes mellitus	206 (32.8%)
Cardiovascular disease	85 (13.5%)
Cerebrovascular disease	21 (3.34%)
Chronic kidney disease	24 (3.8%)
Malignancy	27 (4.3%)
Dyslipidemia	174 (27.7%)
Obesity*	200 (n = 625) (32%)
Pulmonary disease	55 (8.8%)
Dexamethasone**	586 (93.3%)
Laboratory findings (median/IQR)	
WBC count (× 10^3^ cells/µL)	7.7 (5.7–10.6)
Lymphocytes (/µL)	11 (7–16.75)
Platelets (× 10^3^ cells/µL)	187.5 (147–238)
CRP (mg/dL)	85.05 (40.25–153.88)
Creatinine (mg/dL)	0.91 (0.74–1.1)
Neutrophils-lymphocytes ratio	7.5 (4.5–12.9)
Hypoglycemia	59 (9.4%)
ICU admission	374 (59.6%)
Mechanical ventilation	229 (36.5%)
Acute renal failure	67 (10.7%)
Length of stay (median, IQR)	10 (6–18.8)
Mortality	131 (20.9%)

Data are presented as mean ± SD, number (%), and median (IQR)

SD: standard deviation; CV: coefficient of variation; n: number; IQR: interquartile range; CRP: C-reactive protein; WBC: white blood cell; ICU: intensive care unit. Obesity*: data for the calculation of body mass index were not available for all patients. Dexamethasone**: Inhospital dexamethasone administration

The ROC curve fitted to estimate the SD and CV cutoff values associated with mortality identified the values of 44.7 mg/dL for SD and 27.5% for CV. These cutoff values for SD and CV were calculated for this study population, specifically. The patients were subsequently stratified into groups for each study metric defining low vs. high glycemic variability, i.e., SD < 44.7 mg/dL (n = 357) vs. ≥ 44.7 mg/dL (n = 271), respectively, and CV < 27.5% (n = 318) vs. ≥ 27.5% (n = 310), respectively (Table 2). Values ≥ 44.7 mg/dL for SD and ≥ 27.5% for CV were defined as indicative of high glycemic variability.

**Table 2:** Characteristics of the study population stratified by glycemic variability according to standard deviation and coefficient of variation values

	SD < 44.7	SD ≥ 44.7	p	CV < 27.5	CV ≥ 27.5	p
Patients—n (%)	357 (56.84%)	271 (43.15%)		318 (50.63%)	310 (49.36%)	
Age (years)—(mean ± SD)	56.15 ± 16.41	64.18 ± 13.97	< 0.001	55.5 ± 15.9	63.79 ± 14.76	< 0.001
Male gender—n (%)	238 (66.7%)	175 (64.6%)	0.584	211 (66.4%)	202 (65.2%)	0.753
Comorbidities						
Hypertension	158 (44.3%)	170 (62.7%)	< 0.001	141 (44.3%)	187 (60.3%)	< 0.001
Diabetes mellitus	53 (14.8%)	153 (56.5%)	< 0.001	52 (16.4%)	154 (49.7%)	< 0.001
Cardiovascular disease	29 (8.1%)	56 (20.7%)	< 0.001	26 (8.2%)	59 (19%)	< 0.001
Cerebrovascular disease	8 (2.2%)	13 (4.8%)	0.078	6 (1.9%)	15 (4.8%)	0.04
Chronic kidney disease	9 (2.5%)	15 (5.5%)	0.051	4 (2.5%)	20 (6.5%)	0.01
Malignancy	20 (5.6%)	7 (2.6%)	0.065	16 (5%)	11 (3.5%)	0.36
Dyslipidemia	75 (21%)	99 (36.5%)	< 0.001	65 (20.4%)	109 (35.2%)	< 0.001
Obesity*	116 (32.6%)	84 (31.2%)	0.719	104 (32.8%)	96 (31.2%)	0.661
Pulmonary disease	24 (6.7%)	31 (11.4%)	0.038	20 (6.3%)	35 (11.3%)	0.027
Dexamethasone**	324 (90.8%)	262 (96.7%)	0.03	288 (90.6%)	298 (96.1%)	0.05
Laboratory findings (median/IQR)						
WBC count (× 10^3^ cells/μL)	7.4 (5.6–10.2)	7.7 (5.8–11)	0.213	7.4 (5.8–10.3)	7.6 (5.8–10.7)	0.342
Lymphocytes (/μL)	11 (7–17.5)	11 (1–16)	0.076	11 (7–17)	11 (7–16)	0.091
Platelets (× 10^3^ cells/μL)	191 (149–239)	182 (142–235)	0.348	194 (152.8–240)	179.5 (139.8–234)	0.143
CRP (mg/dL)	11.7 (37.9–146)	114.7 (46.8–159.5)	0.211	91.35 (39.1–150.8)	81.5 (41.4–156.1)	0.917
Creatinine (mg/dL)	0.89 (0.74–1.08)	0.93 (0.76–1.19)	< 0.001	0.89 (0.7–1.06)	0.93 (0.77–1.18)	< 0.001
Neutrophils-lymphocytes ratio	7.2 (4.4–12.7)	7.7 (4.7–12.9)	0.46	7.5 (4.4–12.7)	7.55 (4.5–12.9)	0.512
Capillary glucose levels (mean ± SD) (mg/dL)	125.7 (± 16.6)	195.5 (± 47.2)	< 0.001	130.8 (± 31.1)	181.4 (± 49)	< 0.001
Hypoglycemia	21 (5.9%)	38 (14%)	< 0.001	13 (4.1%)	46 (14.8%)	0.01
ICU admission	191 (53.5%)	183 (67.5%)	< 0.001	171 (53.8%)	203 (65.5%)	0.003
Mechanical ventilation	93 (26.1%)	136 (50.2%)	< 0.001	89 (28%)	140 (45.2%)	< 0.001
Acute renal failure	30 (8.4%)	37 (13.7%)	0.035	28 (8.8%)	39 (12.6%)	0.125
Length of hospital stay (median, IQR)	9 (6–16)	12 (7–24)	< 0.001	9 (5–17)	12 (7–22)	< 0.001
Mortality	45 (12.6%)	86 (31.7%)	< 0.001	39 (12.3%)	92 (29.7%)	< 0.001

Data are presented as mean ± SD, number (%), and median (IQR)

SD: standard deviation; CV: coefficient of variation; n: number; IQR: interquartile range; CRP: C-reactive protein; WBC: white blood cell; ICU: intensive care unit. Obesity*: data for the calculation of body mass index were not available for all patients. Dexamethasone**: Inhospital dexamethasone administration

Overall, patients with SD ≥ 44.7 mg/dL and CV ≥ 27.5% were more frequently men and older than those with SD < 44.7 mg/dL and CV < 27.5% (p values = 0.054 and < 0.001). Patients with higher glycemic variability were more likely to present with previous comorbidities (hypertension [p < 0.001], DM [p < 0.001], cardiovascular disease [p < 0.001], chronic kidney disease [p = 0.05] dyslipidemia [p < 0.001], and pulmonary disease [p = 0.038]) than those without comorbidities. Patients with SD ≥ 44.7 mg/dL and CV ≥ 27.5% had higher median serum creatinine level at admission (0.93 mg/dL, IQR 0.76–1.19 mg/dL and 0.93 mg/dL, IQR 0.77–1.18 mg/dL), respectively) than those with SD < 44.7 mg/dL and CV < 27.5% (0.89 mg/dL, IQR 0.74–1.08 mg/dL, p = 0.001 when compared with SD ≥ 44.7 mg/dL and 0.89 mg/dL, IQR 0.7–1.6 mg/dL, p = 0.001 when compared with CV ≥ 27.5%, respectively). The number of lymphocytes and platelets, CRP levels, and N/L values did not differ significantly between the groups. Mortality was significantly higher in the groups with an SD ≥ 44.7 mg/dL (p < 0.001) and CV ≥ 27.5% (p < 0.001) (Table 2).

**Fig. 2:**
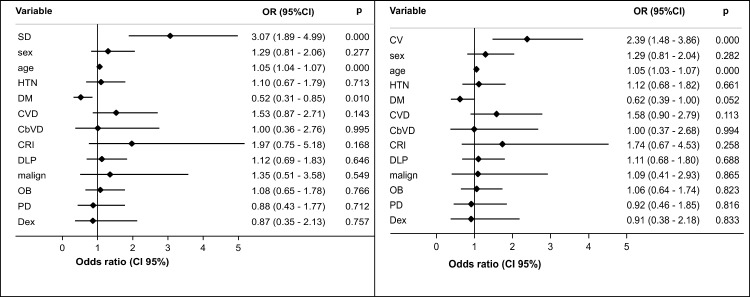
Forest plot of the multivariate association of mortality in patients hospitalized with COVID-19, adjusted for glucose standard deviation and coefficient of variation, sex, age, comorbidities, and use of dexamethasone in hospital. SD: glucose standard deviation; CV: glucose coefficient of variation; OR: odds ratio; 95% CI: 95% confidence interval; HTN: hypertension; DM: diabetes mellitus; CVD: cardiovascular disease; CbVD: Cerebrovascular disease; CRI: chronic renal injury; DLP: dyslipidemia; malign: malignancy; OB: obesity; PD: pulmonary disease; Dex: use of dexamethasone in hospital

In the unadjusted models, the odds of mortality were higher in patients with SD ≥ 44.7 mg/dL (OR 3.22, 95% CI 2.15–4.87) and CV ≥ 27.5% (OR 3.02, 95% CI 2–4.57) than in those with SD < 44.7 mg/dL and CV < 27.5%. The risk of mortality remained high in patients with SD ≥ 44.7 mg/dL and CV ≥ 27.5% after adjusting for age, sex, and the presence of comorbidities (hypertension, cardiovascular disease, diabetes, obesity, cerebrovascular disease, chronic kidney injury, pulmonary disease and dyslipidemia) and use versus no use of corticosteroids during hospitalization (Fig. 2).

The cohort was also stratified according to the absence (n = 422) or presence (n = 206) of DM. Patients with DM were older (p < 0.001) and more likely to present with chronic conditions (hypertension, cardiovascular disease, and dyslipidemia; p < 0.001) than those without DM (Table 3). The mortality rate was higher with SD ≥ 44.7 mg/dL and CV ≥ 27.5% (compared with SD < 44.7 mg/dL and CV < 27.5%) both in the presence and absence of DM (p < 0.001 for all comparisons) (Table 3).

**Table 3:** Characteristics of the study population stratified by the presence or absence of previous diabetes mellitus (DM)

	Entire cohort (n)	Patients without DM	Patients with DM	p values
Patients—n (%)	628 (100%)	422 (67.2%)	206 (32.8%)	N/A
Age (years)—mean (± SD)	59.62 ± 15.9	57.11 ± 16.23	64.75 ± 13.88	< 0.001
Male sex (%)	413 (65.8%)	238 (66.7%)	175 (64.6%)	0.532
Comorbidities				
Hypertension	328 (52.2%)	187 (44.3%)	141 (68.4%)	< 0.001
Cardiovascular disease	85 (13.5%)	40 (9.5%)	45 (21.8%)	< 0.001
Cerebrovascular disease	21 (3.34%)	11 (2.6%)	10 (4.9%)	0.159
Chronic kidney disease	24 (3.8%)	12 (2.8%)	12 (5.8%)	0.078
Malignancy	27 (4.3%)	19 (4.5%)	8 (3.9%)	0.836
Dyslipidemia	174 (27.7%)	82 (19.4%)	92 (44.7%)	< 0.001
Obesity*	200 (n = 625) (32%)	136 (32%)	65 (31.6%)	1
Pulmonary disease	55 (8.8%)	40 (9.5%)	15 (7.3%)	0.452
Outpatient insulin use	18 (2.9%)	0	18 (8.7%)	N/A
Dexamethasone use**	586 (93.3%)	397 (94.1%)	189 (91.7%)	0.308
Outcome				
Hypoglycemia	59 (9.4%)	30 (7.1%)	29 (14.1%)	0.008
Mortality	131 (20.9%)	82 (19.4%)	49 (23.8%)	0.211

Data are presented as mean ± SD, number (%), and median (IQR)

DM: diabetes mellitus; n: number. Obesity*: data for the calculation of body mass index were not available for all patients. Dexamethasone**: Inhospital dexamethasone administration

Most patients were treated with dexamethasone during hospitalization, which could have contributed to the high glycemic variability in patients with and without DM. Both the SD (p < 0.001) and CV (p < 0.001) were significantly higher in this population (Table 4).

**Table 4:** Mortality according to glycemic variability (SD and CV) in patients with and without previous diabetes mellitus

	SD < 44.7 mg/dL		SD ≥ 44.7 mg/dL		p values
	Without DM	With DM	Without DM	With DM	
	n = 266	n = 52	n = 156	n = 154	
Mortality (n = 131)	41 (13.5%)	4 (7.5%)	41 (34.7%)	45 (29.4%)	< 0.001
Mortality*			38 (33%)	43 (29.3%)	0.59

	CV < 27.5%		CV ≥ 27.5%		
	Without DM	With DM	Without DM	With DM	
	n = 304	n = 53	n = 118	n = 153	
Mortality (n = 131)	35 (13.2%)	4 (7.7%)	47 (30.1%)	45 (29.2%)	< 0.001
Mortality*			43 (28.3%)	43 (29.5%)	0.9

Data are expressed as n (%).SD: standard deviation; CV: coefficient of variation. diabetes mellitus (DM)

The comparison was made between the groups SD < 44.7 mg/dL and SD ≥ 44.7 mg/dL and CV < 27.5% and CV ≥ 27.5%. These groups were divided into patients with and without DM, to better illustrate the outcome

^*^Mortality = mortality in patients with SD ≥ 44.7 mg/dL or CV ≥ 27.5% in whom dexamethasone was administered during hospitalization

## Discussion

Our results demonstrated that glycemic variability, assessed as glucose SD ≥ 44.7 mg/dL or CV ≥ 27.5% during hospitalization, was associated with substantially increased odds of mortality among patients with COVID-19 with or without DM. This association persisted even after adjustments for age and the presence of cardiovascular disease and DM. Glucose POCT data were collected for 2–10 days, depending on the patients' length of hospital stay. We found that high glycemic variability influenced the risk of mortality in patients hospitalized for COVID-19.

Mehta et al. concluded that there is no association between serum glucose at admission and outpatient and inpatient control with mortality in COVID-19, but the prevalence of this viral disease in the studied region was very low; therefore, these results cannot be considered for regions with a higher prevalence of COVID-19 [2]. Another retrospective study with only 77 patients demonstrated that an uncontrolled glycemic control (HbA1c ≥ 6.5%) before admission was an independent risk factor associated with in-hospital death in patients with COVID-19 [10]. The association between hyper and hypoglycemia and poor outcomes in hospitalized patients with COVID-19 was demonstrated in a multicenter retrospective study [11]. Atamna et al. demonstrated that increased GV was associated with an increased risk of bacteremia and mortality in patients hospitalized with acute infectious disease, with or without previous DM [5]. Although it is still unclear whether the severity of COVID-19 is influenced by glycemic control, or if glycemic variability can predict mortality and poor outcomes in hospitalized patients, the findings of our study suggest that the latter is likely to occur. This result build on those from retrospective studies that focused on glycemic control and clinical outcomes in patients hospitalized with COVID-19. Previous studies have demonstrated that DM and hyperglycemia could lead to susceptibility and complications of other infectious diseases caused by H1N1, SARS-COV-1, and Middle East respiratory syndrome corona virus [12, 13]. Currently, DM is considered a risk factor for COVID-19 complications and death [14, 15], probably because it leads to oxidative stress and severe glycation with overproduction of advanced glycation end products, and hyperglycemia is reported to increase the secretion of tumor necrosis factor alpha and interleukin 10, which could exacerbate inflammation [14–16].

Bhatti et al. and Singh et al. showed that hyperglycemia at admission or during hospitalization predicts poor outcomes and mortality in patients hospitalized with COVID-19 [3, 17]. The mechanisms explaining this occurrence involve functional impairment of T cells, which plays an important role in the resolution of viral infections. Hyperglycemia increases the intracellular concentration of glucose and mitochondrial proton gradients and releases reactive oxygen species (ROS) [18]. This causes structural modification of T cell receptor proteins and reduces the immune response of T cells via CD3 signaling [18]. High glycemic variability can boost oxidative stress, which can lead to a deficient T cell response, intensifying the severe manifestations of COVID-19 and increasing mortality [16].

In this study, SD and CV were used as the gold-standard metrics to determine glycemic variability, both of which are simple and accurate methods for this assessment [19–21]. Previous studies have demonstrated the impact of glucose variability in hospitalized patients. Singh et al. in a prospective study showed that high glycemic variability is associated with increased mortality among critically ill patients [22]. A retrospective study has demonstrated that in patients hospitalized with other types of acute infections, high glycemic variability was associated with mortality in the ICU and non-ICU settings [5]. Hartmann et al. in a retrospective study, has demonstrated that high variability of fasting plasma glucose was associated with an increased risk of mortality in severely ill COVID-19 patients with acute respiratory distress syndrome [23]. Our study, which focused on patients hospitalized with COVID-19, corroborates these findings. A particularly notable finding was the effect of glycemic variability on mortality risk among patients without DM. Morse et al. reported comparable results in a retrospective study of patients with COVID-19; the authors observed higher mortality in patients with one or more high glucose measurements than in those with all glucose measurements within the normal range [24]. The authors concluded that hyperglycemia was an independent risk factor for mortality in this population. The NICE-SUGAR study, a large, international, randomized trial, demonstrated a 2.6% increase in mortality among critically ill patients receiving intensive insulin therapy (aimed at glucose levels between 81 and 108 mg/dL) compared with those receiving conventional insulin therapies. The increased mortality observed has been attributed to an increased risk of hypoglycemia [25]. These results suggest that the most important factor for mortality risk is not a single glucose measurement but a patient’s glycemic variability.

Current guidelines recommend controlling glucose levels in all hospitalized patients [26, 27]. However, some hospitals often adopt this recommendation only for patients with DM or those who are critically ill. The findings of our study confirm that blood glucose levels must be monitored in all patients with COVID-19 regardless of the presence of comorbidities. We observed that values of SD ≥ 44.7 mg/dL or CV ≥ 27.5% during hospital stay emerged as predictors of mortality, independent of the patients' age or presence of DM and cardiovascular disease. We demonstrated that glycemic variability, not the presence of DM itself, was the main cause of increased mortality risk in patients hospitalized with COVID-19. This may have occurred because patients with DM are more likely to have hyperglycemia and may better endure the deleterious effects of acute hyperglycemia during critical illness, having different biological and/or clinical implications [24, 28, 29]. Another important factor is performance bias, in which patients with DM receive more attention from the medical team in terms of glycemic control to avoid hyperglycemia or hypoglycemia during insulin treatment [29].

Many of our patients were treated with dexamethasone during hospitalization, which could have contributed to the high glycemic variability in patients with and without DM. However, in this study, the association of the influence of dexamethasone treatment on mortality was unclear, and the number of patients who did not use dexamethasone was too low to allow for the presence or absence of this association. Lesniak et al. reported a similar finding, in which the use of corticosteroids in their study worsened glycemic variability by inducing hyperglycemia and insulin resistance [30]. The Randomized Evaluation of COVID-19 Therapy (RECOVERY) trial showed that treatment with dexamethasone reduces mortality in inpatients with COVID-19 requiring oxygen therapy and mechanical ventilation [30, 31]. Lim et al. concluded that dexamethasone had a protective effect in their study population, despite increasing glycemic variability [31].

This study has some limitations. First, it was conducted at a single center (a private hospital in the city of Curitiba); thus, our patient population may be most selected in relation to those assisted in other hospitals. Second, patients with a history of hyperglycemia or hypoglycemia have a greater chance of having their capillary glucose measured more often; thus, the probability of detecting increasing glycemic variability in these patients was higher. Important strengths of our study include: first, it is real-world evidence data with a prospective design; second, data collection was monitored in real time by the authors; third, all patients were included consecutively; fourth, measurements were taken in all patients with glucose levels at least three times daily by POCT using bedside glucometers, supporting an accurate estimation of glycemic variability; and fifth, we performed the evaluation of glycemic variability using two different simple metrics (SD and CV).

## Conclusions

Glucose monitoring is recommended in all patients hospitalized with COVID-19, including those without DM. We found that glycemic variability was indicative of poor prognosis in patients hospitalized with COVID-19.

Glycemic variability associated independently with mortality in patients with and without DM who were hospitalized with COVID-19, early intervention to improve glucose control could improve patient outcomes.

## Data Availability

Not applicable.

## Abbreviations

COVID-19: Coronavirus disease 2019
CI: Confidence intervals
CRD: Chronic respiratory diseases
CRP: C-reactive protein
CV: Coefficient of variation
CVD: Cardiovascular disease
DM: Diabetes mellitus
GV: Glucose variability
ICU: Intensive care unit
IQR: Interquartile range
OR: Odds ratio
N: Number
POCTs: Point-of-care capillary glucose tests
RT-PCR: Reverse transcription polymerase chain reaction
SARS-CoV-2: Severe acute respiratory syndrome coronavirus 2
SD: Standard deviation
WBC: White blood cell
